# Improving Quality Metrics with a Day-only Skin Abscess Protocol: Experience from Australia

**DOI:** 10.1007/s00268-023-06941-6

**Published:** 2023-02-22

**Authors:** Crystal Li, Peter Nguyen, Parul Garg, Helen Pham, Kerry Hitos, Tony Pang

**Affiliations:** 1 Surgical Innovations Unit, Westmead Hospital, Cnr Hawkesbury and Darcy Road, Westmead, NSW 2145 Australia; 2 Westmead Research Centre for Evaluation of Surgical Outcomes, Department of Surgery, Westmead Hospital, Sydney, Australia; 3 Westmead Clinical School, Faculty of Medicine and Health, The University of Sydney, Sydney, Australia; 4 Department of Surgery, Westmead Hospital, Sydney, Australia

## Abstract

**Background:**

Skin abscesses are a common emergency presentation often requiring incision and drainage; however, issues with theatre access lead to delays in management and high costs. The long-term impact in a tertiary centre of a standardised day-only protocol is unknown. The aim was to evaluate the impact of day-only skin abscess protocol (DOSAP) for emergency surgery of skin abscesses in a tertiary institution in Australia and to provide a blueprint for other institutions.

**Methods:**

A retrospective cohort study analysed several time periods: Period A (July 2014 to 2015, *n* = 201) pre-DOSAP implementation, Period *B* (July 2016 to 2017, *n* = 259) post-DOSAP, and Period *C* (July 2018 to 2022, *n* = 1,625) prospectively analysed four 12-month periods to assess long-term utilisation of DOSAP. Primary outcomes were length of stay and delay to theatre. Secondary outcome measures included theatre start time, representation rates and total costs. Statistical analysis using nonparametric methods was used to analyse the data.

**Results:**

There was a significant decrease in ward length of stay (1.25 days vs. 0.65 days, *P* < 0.0001), delay to theatre (0.81 days vs. 0.44 days, *P* < 0.0001) and theatre start time before 10AM (44 cases vs. 96 cases, *P* < 0.0001) after implementation of DOSAP. There was a significant decrease in median cost of admission of $711.74 after accounting for inflation. Period *C* reported 1,006 abscess presentations successfully managed by DOSAP over the four-year period.

**Conclusion:**

Our study demonstrates the successful implementation of DOSAP in an Australian tertiary centre. The ongoing utilisation of the protocol demonstrates the ease of applicability.

**Supplementary Information:**

The online version contains supplementary material available at 10.1007/s00268-023-06941-6.

## Introduction

Skin abscesses are a common emergency presentation requiring surgical consultation and often operative management [1]. Incision and drainage of the skin abscess either under local or general anaesthesia remain the gold standard of treatment [2]. Often, this is performed in an operating theatre setting; however, this may lead to high treatment costs due to theatre access issues or unnecessary postoperative stay.

One solution is to perform abscess drainage as a day-only surgery (i.e., same-day discharge). Day-only surgery has been successfully implemented for elective surgery patients for operations such as hernia repairs and laparoscopic cholecystectomies. There is strong evidence to support that patient safety is maintained with no significant differences in patient mortality or complication rates [3]. More recently due to the COVID-19 pandemic, there has been an increasing selection of emergency cases that have been adapted to fit this model of care. Conaghan et al. first showed a statistically significant decrease in hospital length of stay (0 days vs. 2 days, *P* < 0.001) and admission costs in patients presenting with acute surgical conditions such as acutely painful hernias, superficial abscesses and thrombosed haemorrhoids [4]. Patient-reported outcomes in elective cases have also demonstrated significant improvement under this model of care [5].

Although there has been some success reported from the implementation of fast-track surgery for abscesses in Australia in a single institution, the long-term system and financial impact in a large tertiary centre of a standardised day-only protocol are not well defined [6]. We hypothesise that a well-designed day-only abscess protocol would have impact not only on patient outcomes and cost-savings, but changes to the culture of case prioritisation and long-term surgical practice within the institution. The aims of this study are to evaluate the impact of implementation of a day-only protocol for emergency surgery of skin abscesses in a tertiary hospital in Australia and provide a blueprint for other institutions wishing to implement such a protocol.

## Methods

This is a retrospective cohort study comparing patient outcomes before the protocolised care of patients with skin abscesses and those managed after the implementation of the day-only skin abscess protocol (DOSAP).

Patients from three time periods were reviewed and analysed. Period *A* is from July 2014 to July 2015 correlating to prior to DOSAP implementation. Period *B* is from July 2016 to July 2017 correlating to following introduction of DOSAP. Period *C* is from July 2018 to July 2022 correlating to a period to assess long-term adherence to protocol. Patients from Periods *A* to *B* were compared in detail, as they represent the most comparable group of patients due to their close temporal relationship. The period between July 2015 and July 2016 was excluded as this period represented a transition period where some aspects of DOSAP were instituted but not formally implemented. DOSAP was formally implemented in early 2016. Patients from Period *C* were additionally analysed to explore the durability of the clinical practice changes brought about by DOSAP.

### The day-only skin abscess protocol (DOSAP)

The full protocol and its implementation are provided as supplementary material. The full protocol describes patient selection, treatment (including antibiotic selection and operative management), as well as processes to streamline the logistics of readmission for surgery. The following briefly describes the protocol.

#### Patient selection

**Fig. 1:**
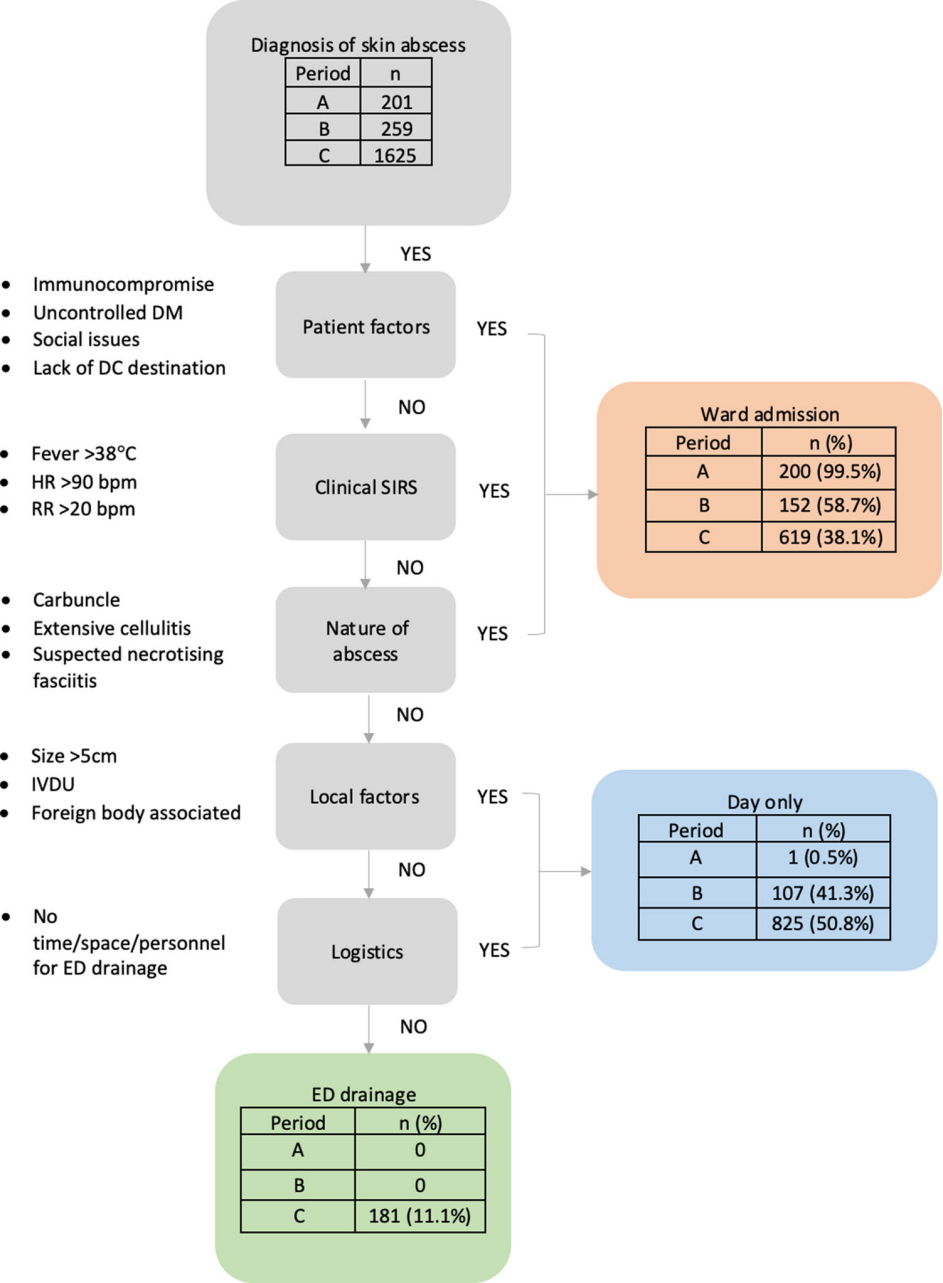
Flow chart summarising patient selection for DOSAP. Patients with a diagnosis of skin abscess are deemed suitable for drainage in the emergency department or in day-only theatre based on a process of exclusion. Patients are excluded on the basis of patient factors (immunocompromise, poorly controlled diabetes mellitus, social issues such as no suitable discharge destination), clinical factors (overt systemic inflammatory response), and the nature of the abscess (carbuncles, extensive cellulitis, or suspected necrotizing soft tissue infection). Those with abscesses satisfying DOSAP exclusion required ward admission and inpatient operative management. If the patient had none of these exclusion factors, they were considered for either drainage in the emergency department or as day-only surgery. Emergency department (ED) drainage is preferable, as long as local abscess factors or logistic factors did not make ED drainage unfeasible. Bpm—beats/breaths per minute, DC—discharge, DM—diabetes mellitus, ED—emergency department, HR—heart rate, IVDU—intravenous drug use, RR—respiratory rate, SIRS—systemic inflammatory response syndrome

Patient selection is based on a process of exclusion (Fig. 1). Those with patient factors predicting poor outcome, systemic inflammatory response syndrome (SIRS) or certain local abscess characteristics were excluded from day-only management. These patients were managed by drainage within the operating theatre setting *AND* stay overnight in a surgical ward bed. Those eligible for day-only management were further stratified into those who are suitable for day-only theatre drainage or day-only emergency department (ED) drainage, depending on local or logistic factors.

#### Bed management

Bed allocation for patients who are eligible for day-only theatre drainage depends on the time of the day. Generally, drainage is organised either on the same day (if the patient was assessed within office-hours and there is available time on the emergency theatre list) or organised as the first case for the following morning. The latter patients are discharged home, with appropriate instructions to fast and represent the following day for surgery. After drainage, all patients were aimed to be discharged after appropriate post-operative observation on the same day.

#### Management

Antibiotics were indicated in patients with systemic symptoms (temperature > 38 °C, heart rate >90 bpm or systolic blood pressure <90 mmHg) or cellulitis. Day-only abscess patients presenting with cellulitis were discharged with oral antibiotics, and those fulfilling SIRS criteria were commenced on intravenous antibiotics. Patients were instructed to follow up with their general practitioner, if required, in three days to reassess the need for antibiotics, for example, in the case of ongoing cellulitis. A recommended abscess drainage procedure is included in the protocol, although this was not prescriptive. Wound packing was discouraged.

### Analysis

Data analysis included demographic characteristics, clinical and management data, logistical information such as hospital length of stay, wait times and cost data.

The primary outcome measures are hospital length of stay and patient wait times. Secondary outcome measures included theatre start time, representation rates and total costs. Costings data were retrieved from the relevant hospital department; thus, it relies on routine administrative collection. In costs between years, inflation was accounted for by the Australian Consumer Price Index. Costs included both the costs for the ED and the admission (for surgery) encounters.

All data were entered and analysed using IBM SPSS Statistics version 26.0 for Windows (IBM Corp., Armonk, NY, USA). Data normality and distribution were evaluated and summarised using descriptive statistics. Continuous data were presented as the means with their standard deviations or medians and interquartile range (IQR). Pre- and post-protocol patient characteristics were directly compared. For this, continuous data were compared using the Student’s t test, Fisher’s exact test, Mann–Whitney *U* test or Kruskal–Wallis test. All tests were two tailed with statistically significant differences considered at the *P* < 0.05 level.

## Results

During Periods *A* (pre-DOSAP) and *B* (post-DOSAP), a total of 460 cases of skin abscesses were referred for surgical treatment. There was no significant difference in demographics between the two groups (Table 1). Apart from the mean blood sugar being statistically, but not clinically, significantly higher (5.5 mmol/L vs. 5.2 mmol/L), there were no significant differences between patients of the two groups. Interestingly, the patients in Period *B* were more likely to have a longer duration of symptoms and were more likely to have had previous incision and drainage prior to presentation.

**Table 1:** Demographic and clinical details of the participants’ pre- and post-implementation of DOSAP

Variable	Pre-protocol (Period *A*) (*n* = 201)	Post-protocol (Period *B*) (*n* = 259)	*P* value
	*n* (%) or median (IQR)		
Male sex	116 (58%)	165 (64%)	0.191
Age (years)	34 (25–46)	33 (24–46)	0.272
*Clinical characteristics*			
Diabetes mellitus	30 (15%)	46 (18%)	0.458
Immunocompromised	9 (4.5%)	11 (4.2%)	0.886
Temperature (°C)	37 (36–37)	37.0 (36–38)	0.375
Heart rate (per minute)	87 (77–102)	96 (83–105)	0.257
Respiratory rate (per minute)	16 (16–18)	16 (16–18)	0.181
White cell count > 12 × 10^9^	77 (39%)	88 (37%)	0.676
Abscess size (cm)	4 (3–7)	4 (3–5)	0.692
Blood sugar level	5.2 (5–6)	5.5 (5–7)	**0.014**
Multi-resistant *Staphylococcus aureus* (MRSA)	9 (4.5%)	11 (4.2%)	0.886
*Management characteristics*			
Ultrasound	15 (8%)	30 (12%)	0.141
Incision and drainage prior to presentation	46 (23%)	86 (33%)	**0.015**
Duration of symptoms (days)	4 (3–7)	5 (3–7)	**0.001**

Bold values indicate statistical significance (*P* < 0.05)

There was a significant decrease in ward length of stay (1.25 days vs. 0.65 days) and delay time to theatre (0.81 days vs. 0.44 days) after implementation of the protocol (Table 2). This decrease in delay time to theatre was likely influenced by the earlier start times in theatre. Most cases started earlier in the day with a significant increase in theatre times starting before 10AM (21.9% vs. 37.1%) and before midday (38.3% vs. 55.6%) after implementation of the protocol. The median admission length of stay in those requiring inpatient drainage in Period A was 1.48 days (IQR 0.93–2.08) and in Period B was 1.12 days (IQR 0.76–2.09) (*P* = 0.035). There was no significant difference in wait time in ED and representation rates between the two groups. Following implementation of the protocol, there was also a significant decrease in median cost of admission with a reduction of $711.74 after accounting for inflation (Table 3).

**Table 2:** Pre-protocol and post-protocol outcomes

Outcome	Pre-protocol	Post-protocol	*P* value
Day-only rate (%)	0.5%	41.3%	**< 0.0001**
Median total ward length of stay in days (IQR)	1.25 (0.88–1.93)	0.65 (0.31–1.45)	**< 0.0001**
Median total delay to theatre in days (IQR)	0.81 (0.33–1.05)	0.44 (0.29–0.75)	**< 0.0001**
Median wait time in ED in days (IQR)	0.23 (0.16–0.28)	0.22 (0.15–0.33)	0.514
Theatre start time before 12 pm	77 (38.3%)	144 (55.6%)	**0.0001**
Theatre start time before 10 am	44 (21.9%)	96 (37.1%)	**0.0001**
Representation rates	21 (10.6%)	27 (10.4%)	0.965

Bold values indicate statistical significance (*P* < 0.05)

**Table 3:** Pre-protocol and post-protocol effect on cost

Outcome	Pre-protocol	Post-protocol	Reduction	*P* value
Median cost of admission (IQR)	$4314.40 ($3376.80–$5882.56)	$3602.66 ($2886.71–$4885.22)	$711.74 ($490.09–$997.33)	**< 0.0001**

Bold value indicates statistical significance (*P* < 0.05)

**Fig. 2:**
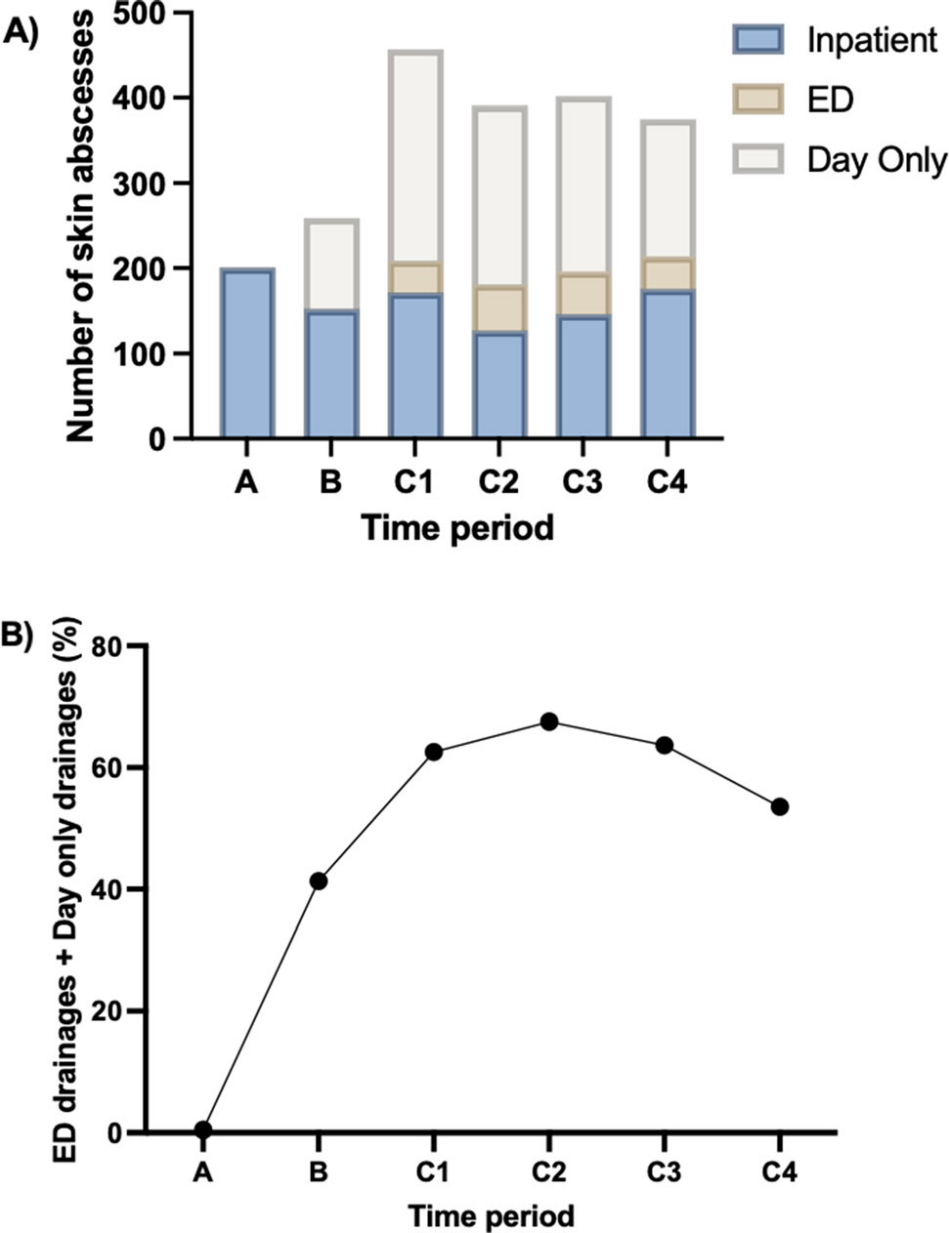
Management of skin abscesses over time. Panel (**a**) illustrates the number of skin abscess patients’ instances over time, which were managed as inpatients, as ED drainages or as day-only patients. Panel (**b**) illustrates the proportion of patients successfully managed without inpatient admission (i.e. ED drainage or day-only drainage). In each plot, the x-axis represents the study periods, as described in the text. Period *A*: July 2014–June 2015. Period *B*: July 2016–June 2017. Period *C*1: July 2018 − June 2019. Period *C*2: July 2019 − June 2020. Period *C*3: July 2020 − June 2021. Period *C*4: July 2021 − June 2022

Period *C* evaluated ongoing utilisation of the DOSAP over a four 12-month periods. Periods *A* and *B*, respectively, reported 1/201 and 107/259 skin abscess presentations appropriate for management under the DOSAP. On the other hand, Period *C* showed that the DOSAP was consistently utilised in management of skin abscesses. Annually, from July 2018 until July 2022, 286/457 (62.3%) skin abscess presentations, 264/391 (67.5%) skin abscess presentation, 256/402 (63.4%) skin abscess presentations and 200/375 (53.3%) skin abscess presentations were considered appropriate for management under DOSAP with either day-only drainage or drainage in the emergency department (Fig. 2).

## Discussion

This study evaluated the impact of the implementation of DOSAP in an Australian setting. We found that DOSAP significantly reduced hospital length of stay and delay time to theatre without increasing readmission rates. We also found it altered the prioritisation of abscess cases, resulting in operations occurring earlier in the day. Ultimately, these changes led to a significant reduction in total hospitalisation costs. Most importantly, we also demonstrated the durability of clinical practice change, as the day-only management rate continued to increase three years after initial implementation.

While skin abscesses constitute one of the least acute surgical presentations, EDs have faced significant strain related to an increase in presentations of skin abscesses [7]. The increasing burden associated with skin abscesses is reflected in our data, which demonstrated that abscess presentations increased from 201 cases per year in Period A to between 375 and 457 cases per year in Period C. Thus, there is a strong need for surgical departments to work collaboratively with EDs to optimise flow of patients to prevent overcrowding and overworking.

In implementing DOSAP, we utilised the Clinical Practice Improvement framework recommended by the Clinical Excellence Commission [8]. We set up a working group comprising all major stakeholder representatives, including ED, surgery, perioperative services, and medical administration. The resulting protocol thus represents more than just the most efficient way to manage skin abscesses, but rather the most efficient way to manage skin abscesses that works with all stakeholders. At the implementation stage, education of staff, especially the junior medical staff, on the protocol was an important factor in its success. To maintain staff awareness and to monitor effectiveness, prospective data collection and audit of day-only rates were continued after implementation. We believe that all of the above factors contributed to the long-term success of DOSAP at our institution.

While not explored in this study, one key motivator to DOSAP is the improvement of patient experience. Unnecessary delays, uncertainty in timing of surgery and repeated fasting in preparation for surgery impact patient experience. In our study, implementation of DOSAP reduced wait time to theatre by almost half a day. It has been shown in the literature that cancellations and delay to theatre are associated with poorer levels of patient satisfaction and experience [9] and that extended periods of fasting were perceived by patients as distrust in the organisation of the procedures and as poor communication from health professionals [10].

A second motivator to DOSAP is cost. In Period C, with on average 406 presentations and costs per patients of between about $3,000 and $6,000, the annual costs of managing skin abscesses exceed $2 million. With an average day-only rate of 51%, the costs of admission decreased by approximately $700. Thus, with increasing utilisation of day-only management, potential cost-savings could be even greater. Some studies reporting cost-savings of more than 300% in day-only laparoscopic cholecystectomy models of care [11]. In addition to monetary savings, there are also savings in bed space utilisation, with follow-up improvements on hospital efficiency, which is difficult to quantify.

The implementation of DOSAP also appeared to trigger a cultural change in the management of skin abscesses. One key finding of this study is the durability of the clinical practice change from DOSAP over a four-year period after implementation. While short-term outcomes have previously been reported [6], no previous study has documented the durability of such a clinical practice change. The second aspect to changing the culture of practice relates to the prioritisation of skin abscess cases. This was reflected in the increase in proportions of theatre start time before 10AM and midday. Traditionally, incision and drainage of skin abscesses were triaged on theatre lists after major cases of higher priority. In the setting of under-provisioned emergency theatre time, such practice leads to a vicious cycle of delays. Indeed, the longest wait for theatre was 6.34 days prior to DOSAP implementation (which decreased to a maximum of 3.14 days after DOSAP). Implementation of the DOSAP allowed for skin abscess procedures to occur either on the same day as presentation (after liaising with the duty anaesthetist) or a planned booking as the first case on the emergency theatre list the following day. While not directly demonstrated in this study, this anecdotally led to improved patient flow and theatre case planning. Previous studies have demonstrated cost-savings with ‘AM’ prioritisation of cases with day-only success rates [12]. Procedures performed earlier in the day allow for shorter fasting periods, as well as early postoperative review and timely in-hours discharge.

One aspect, which was not examined in this paper, is non-inpatient costs of treatments. DOSAP or similar standardised protocols may reduce such costs through the promotion of best practices. An example is the need for wound packing. Traditionally, packing with ribbon gauze was performed to keep the abscess cavity open to allow for healing by secondary intention; however, literature now suggests the reduced need for packing [13]. This reduces the burden on community nurses or primary care providers who were required to review these patients for several days post-operatively. Another example is the rational use of antibiotics in abscess cases. Reduction in unnecessary antibiotic use reduces antibiotic patient complications, costs of antibiotics, as well as minimises the impact on the development of antibiotic resistance in the community.

Our study is not the first study to attempt to change the status quo in management of skin abscesses. Harris et al. described the successful establishment of a clinic, which transitioned the management of soft tissue infections in the intravenous drug user (IVDU) population [14]. The clinic was found to be cost and clinically effective. Despite the similarities, there are clear differences between their approach and that of the present study: their study included all soft tissue infections (skin abscesses being a subset of this), only involved IVDU patients, and also provided non-surgical services such as substance abuse counselling. These differences highlight the need for appropriate stakeholder engagement to establish a service structure which suits the local needs of all stakeholders to ensure successful clinical implementation and durable changes in practice.

The major limitations to our analysis are that this is a retrospective study over different time periods, and so the results may be confounded by non-treatment factors. However, unlike previous studies, this is the first paper that has followed the long-term utilisation of a protocol with prospective data collection of four years. Finally, it is interesting to note that there has been an increase in the numbers of patients presenting with skin abscesses between Period B and C. As the prospective patient identification schema utilised for this study has not changed, it is unlikely to be attributable to patient selection bias. While it is possible that the implementation of DOSAP increased ED referrals for skin abscesses, however, this is unlikely as DOSAP only applied once the patient had been referred to the Acute Surgical Unit. Thus, DOSAP is unlikely to influence ED referral patterns.

## Conclusion

Our study demonstrates the successful implementation of DOSAP in an Australian tertiary centre. The durability of this protocol over time demonstrates the ease of applicability. We hope that our protocol and experience would help other institutions successfully implement similar protocols.

## Supplementary Information

Below is the link to the electronic supplementary material.

Supplementary file1 (DOCX 371 kb)
