# Supplementary material for: Improving Quality Metrics with a Day-only Skin Abscess Protocol: Experience from Australia
Source: World J Surg. 2023 Feb 22;47(6):1486–92. doi: 10.1007/s00268-023-06941-6 (PMC9946283; doi:10.1007/s00268-023-06941-6)
Supplement: Supplementary file 1 — Supplementary file1 (DOCX 371 kb) [file 268_2023_6941_MOESM1_ESM.docx]

Skin abscess protocol – Version 4

Last updated Jan 2022

# Introduction

The aim of this protocol is to improve the efficiency of management of patients with skin abscesses, a common presentation to Westmead Hospital. Ideally, this protocol would promote efficient cooperation amongst ED, ASU and anaesthetic staff. We anticipate that the majority of patients presenting with skin abscesses to the ED would be able to be managed without requiring overnight admission to hospital - either through drainage in the ED or as day only cases using this protocol.

It is important to note that there is little evidence in the literature to guide “optimal” management of these patients. Nonetheless, the application of a standardised management protocol will lead to decreased variations in care and hopefully improved outcomes in terms of both reduced length of stay and improved patient experience.

# Overview of protocol

The Skin Abscess Protocol aims to standardise and streamline the management of all patients with cutaneous abscesses presenting to the ED. The skin abscess protocol can be broken down into the following key components:

- - 1. Determine the appropriate admission type
    2. Arrange appropriate investigations and pre-drainage management
    3. Admission of patient to appropriate ward
    4. Documentation in EMR and theatre booking procedure
    5. Drainage procedure - ED drainage and operating theatre drainage
    6. Post-drainage follow-up care

Despite its apparent complexity, actually one will find that the criteria outlined reflect our usual clinical practice. The only difference is that it is explicitly spelled out in this documentation to minimise practice variation.

# Part 1 - Determine the appropriate management type (Figure 1)

This is summarized below in figure 1. Essentially one needs to consider nature of abscess, patient factors, local factors and logistics. Essentially it is process of exclusion where the most favourable patients get ED drainage and the least favourable patients get ward admissions with OT drainage.


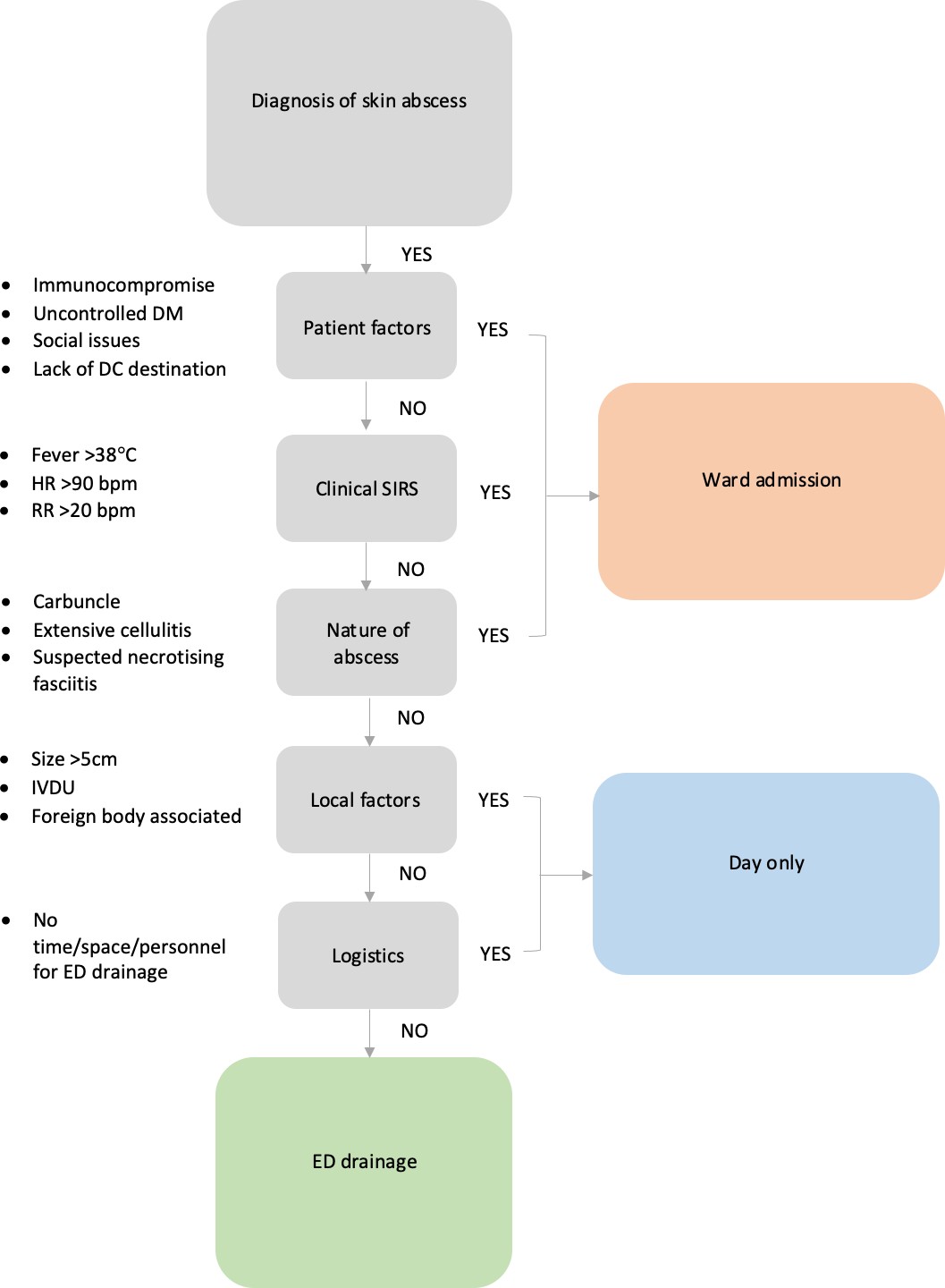


Figure 1 - Management type selection flowchart

# Part 2 – Appropriate investigations and pre-drainage management (Antibiotics)

## Investigations (Figure 2)

- - 1. Finger prick BSL should be performed for all patients
    2. Blood tests - FBC, EUC - should be arranged for patients with any systemic factors excluding them from management in ED
    3. Imaging - Ultrasound if potential vascular involvement (cubital fossa or groin) or if aetiology due to IVDU or if concern regarding foreign body


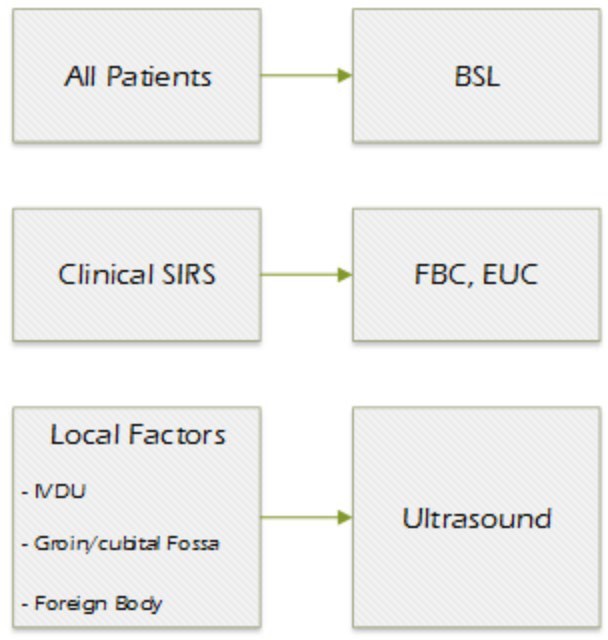


Figure 2 - Investigations flow chart

## Antibiotics selection (Figure 3)

Based upon the following principles:

- - 1. If patient has neither systemic symptoms or cellulitis, antibiotics is **not** required.
    2. If there is non-spreading cellulitis without systemic symptoms, the patient can be discharged on oral antibiotics (and return for theatre the next day)
    3. If SIRS criteria is satisfied, commence intravenous antibiotics (and patient should be admitted).
    4. After drainage: there is good evidence in the literature to suggest that antibiotics is **not** required post drainage of abscesses. The patient should see the GP in 3 days and if cellulitis does not settle then should be commenced on appropriate oral antibiotics.


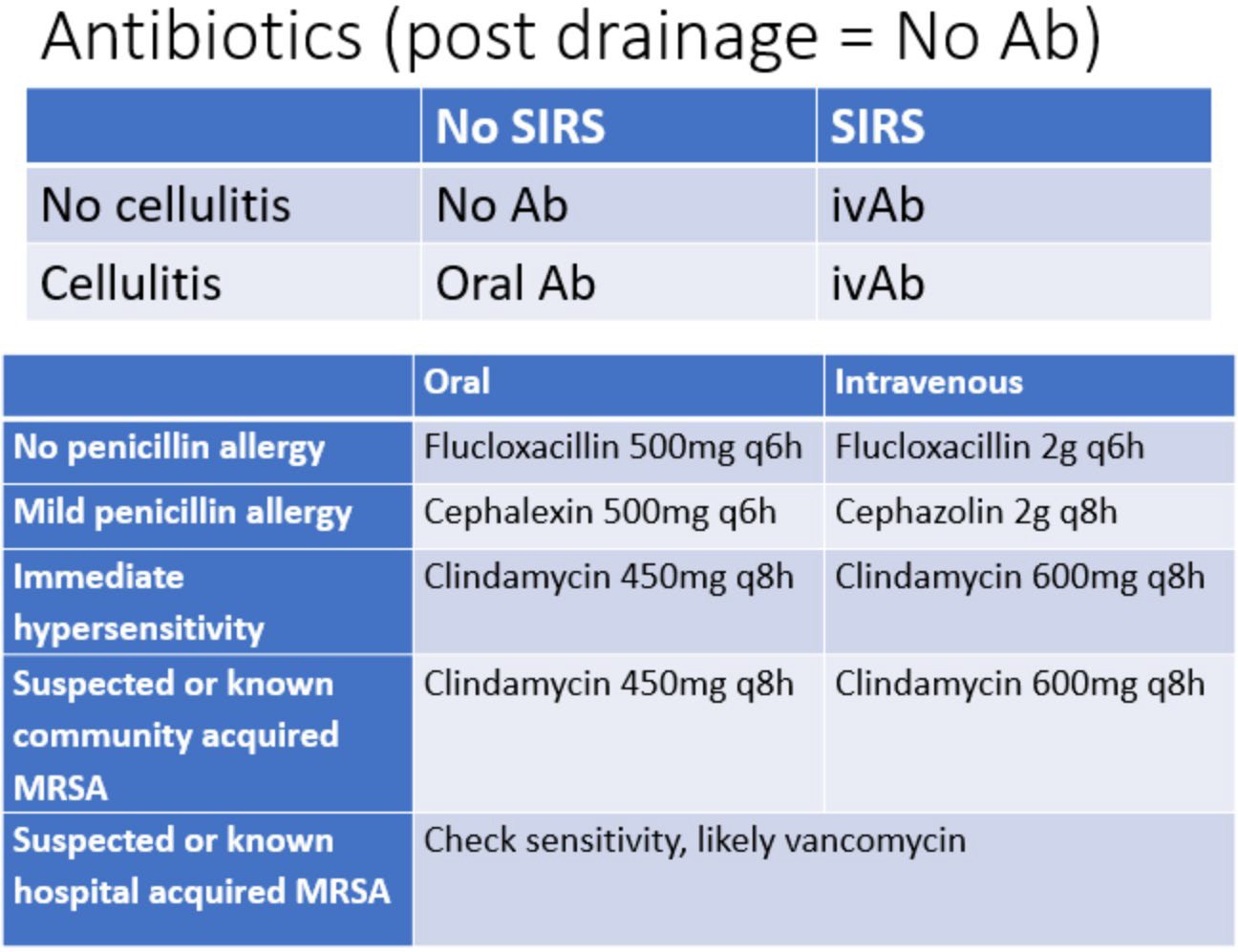


Figure 3 - Antibiotic selection and dosing table

## Admission (Figure 4)

For patients who are suitable for day only management pathway the admission type is based on a Day of the week of presentation (weekday vs. weekend) and Time of day at presentation.

| **Today/tomorrow is:** | **MN-7am** | **7am-5pm** | **5pm-MN** |
| --- | --- | --- | --- |
| **A weekend/public holiday** | Ward admission. Drain and discharge on same day* | Ward admission. Drain and discharge on same day.** | Call PFU to arrange bed for next day. Ask patient to come back to ED 7am |
| **A Weekday** | Ward admission. Drain and discharge on same day* | Day only ward today / next day** | Day only ward next day |

* Unless patient prefers to go home and come back.

** Call 8460 – if can be done realistically by 1800 on the same day, then admit to Day only. If not, arrange for Day Only bed for the next day

Figure 4 - admission type based on day of week and time of day

- 1. **Steps to arrange the appropriate bed are as follows**

1. Next day day-only drainage –
   1. Sunday-Thursday (i.e., next day is Mon-Fri):
      1. If in hours– Call **Day only NUM**
      2. If out of hours (or Sunday) – Call **ADON**
   2. Friday/Saturday (i.e., next day is Sat or Sun or Public Holiday):
      1. If in hours – Call ***Patient Flow Unit***
      2. If out of hours – Call **ADON**

*These patients would be generally allocated a bed as follows: Red patients: K8a*

*Green patients: B3a*

***NOTE:***

***For patients arriving on a weekend/public holiday, please advise patient to present at 7am to ED fasted (Make sure you give the patient a letter addressed to the ED ward clerk!)***

- - 1. Patient will present to ED, get “admitted” on the system by the ward clerk
    2. Patient will then be directed to go to the appropriate ward.

1. Same day DO – **only** if Patient is ready for OT before 1800 (fasted) **and** OT has time to perform drainage by 1800 (call Duty anaesthetist on 8460 to check):
2. Arranged bed with Day only NUM if in hours
3. Surgical short stay (preferred) or Ward Bed (if out of hours)

## Complete appropriate admission paperwork

1. ED drainage –

### Consent

- 1. Admission notes using **abscess protocol note**
  2. Complete the “operation note” using **ED abscess drainage template.**

1. Next day DO admission –

### Consent and Recommendation for Admission (RFA)

- 1. Complete clinical notes on Powerchart using **abscess protocol note**
  2. Complete on-line **Greensheet** and call 8460. Write “** DO abscess protocol **” on Greensheet comments section
  3. **Provide information** to patient:
     1. Give patient information sheet
     2. Advise patient to have no food from midnight and can have clear fluids up until 0600 the following day.
     3. Advise patient to return the following day at 0700 to the Day Only ward
     4. If they can’t speak English, come with someone who can so as to facilitate administrative tasks (like filling forms etc)
     5. Advise that the patient will need to organise pick-up in the afternoon.

1. Next day Surgical Short Stay admission (ie, next day is weekend or public holiday):
   1. Same as (b) above except that you also need to give the patient a letter for the ED ward clerk
2. For all other admission types (Ward/EDO/Same day DO)

### Consent

- 1. Complete clinical notes on Powerchart using **abscess protocol note**
  2. Complete on-line **Greensheet** and call 8460.

*Explanatory notes:*

- The reason for the schedule above is that DO beds operate only Mon-Fri, thus, alternative postoperative beds would need to be arranged over the weekend.
- MN-7am – On Mon-Fri, patient should be admitted to save the patients the inconvenience of going home only for a few hours (although give patient options).
- 7am – 5pm –
  - Mon-Fri – 8460 and Day Only ward should be contacted – if case can be realistically done before 6pm and the patient is ready (ie, fasted), then proceed to DO management. Otherwise discharge and manage as DO case the following day.
  - On Sunday, patient may either be admitted (if OT time allows) or discharged to return on Monday for DO management.
- 5pm – MN –
  - Most patients can be discharged and returned the following day for DO management.

# Additional notes on drainage procedures, patient information and post-procedure care

## Drainage in the ED

### Procedure

- - 1. Local anaesthetic patch (eg, EMLA or AnGel) for half hour
    2. Local infiltration of xylocaine with adrenaline (We have to accept the fact that LA does not work very well in the acidic environment associated with pus, although it is nonetheless better than nothing)
    3. Analgesia with methoxyflurane (“green whistle”)
    4. Preparation with antiseptic solution and drape
    5. **Circular incision** over area of maximal fluctuance just large enough to establish adequate drainage (usually >5-10mm)
    6. Swab taken of pus
    7. Breakdown of loculations, either with

1. Finger
2. Piece of gauze wrapped around tip of forceps/small haemostat
   - 1. Irrigate with saline x30 mL under some pressure (10mL syringe with blunt drawing up needle)
     2. If bleeding, pack with saline soaked gauze ribbon (to be removed by patient next day). If not bleeding, no packing required
     3. Dress with non-occlusive dressing (Usually combine pad secured with 1 or 2 pieces of Micropore tapes)

### Documentation

Use the standard Powerchart operation report for operation note.

### Discharge

1. **Discharge summary** as per usual
2. **Simple analgesia** prn (Panadol/ibuprofen/panadeine as appropriate)
3. Patient information sheet (See Appendix)

### Follow-up

1. GP follow-up in 3 days if required

## Drainage in the OT

### Procedure

- - 1. General Anaesthetic
    2. Position as appropriate, preparation and drape
    3. Circular incision over area of maximal fluctuance just large enough to fit the tip of a finger for effective breakdown of loculations

1. Any necrotic overlying skin should be included in the incision regardless of size (eg, carbuncles may require excision of substantial overlying skin)
   - 1. Swab taken of pus
     2. Breakdown of loculations with finger dissection and finger exploration for extensions.
     3. Irrigate with copious saline, may use hydrogen peroxide to assist haemostasis
     4. If haemostasis is a problem, pack with saline-soaked gauze ribbon of appropriate size. Drains should not be placed unless in exceptional circumstances. If no haemostasis problem, no packing is required.
     5. Dress with a non-occlusive dressing (a combine pad secured with micropore tapes)

**Postoperative orders**

### No antibiotics are required postoperatively unless cellulitis, abscess >5cm or immunosuppression.

Simple analgesics such as paracetamol, NSAIDs should be prescribed.

## Notes on the procedure

Like many technical aspects of surgical practice, there is little evidence to suggest one management is superior to another, and therefore the exact technique depends on personal preference.

However, there is a need for standardisation of technique – one which is safe and effective. Standardisation of technique helps to improve education and training, and fosters effective management of patients by the entire team.

I have found the technique described above to be safe and effective. Whilst many types of incisions have been described (cruciate, linear, elliptical etc), the theoretical advantages of a circular incision is that (1) it is not a wound which lends itself easily to spontaneous closure and therefore theoretically minimises the risk of re-collection; and (2) wound contraction eventually converts the wound into a small dimple which creates an acceptable cosmetic outcome. **Clearly, this is an “evidence-free” zone.**

Traditionally, many abscesses are packed after drainage. The principle of packing is to prevent the premature closure of the wound/tract leading to re-collection. The use of packing therefore in the context of effective drainage is purely for haemostasis. The packing can therefore be removed prior to discharge or by the patient the next morning with no need for continued packing. Drains should be used only in exceptional circumstances (eg, very deep or large cavities or cavities of irregular shape requiring multiple areas of drainage).

## Planning of theatre utilisation by ASU team

Skin abscess drainages should fit into the pre-existing model of utilisation by the ASU team. Currently, the ASU team generally starts any substantial cases at 0830**-**0900 so as to allow some time for the registrar to do a ward round. If skin abscess cases are present, we propose that this should be started at 0830, knife to skin. These cases should be competently handled by the ASU SRMO which will still allow the surgical registrar to complete the morning ward round prior to the more substantial cases to start at 0900. This will lead to the most efficient use of theatre and staffing and minimise late theatre starts. Furthermore, patients are then less likely to wait an entire day fasted for the incision and drainage procedure.

## Discharge

- - 1. Criteria for discharge:

1. Systemically well
2. Minimal cellulitis
3. Pain controlled
4. Appropriate discharge destination
   - 1. Nurse to remove packing on discharge (for EDO patients – for DO patients, patient should be instructed on how to remove pack the following morning)
     2. At discharge, the following must be provided:
5. Patient Information sheet (see Appendix 1) – key points should be verbally explained by the nurse discharging the patient
6. Analgesia – paracetamol + ibuprofen if no contraindications
7. Patients should not be discharged on antibiotics unless there is cellulitis/size>5cm/immunosuppression.

## Follow-up

All patients should be given advice to see a GP in 3 days if required or if they have concerns ASU clinic follow-up is reserved for:

- - 1. Patients re-referred after review by GP
    2. Recurrent pilonidal abscess
    3. Recurrent perianal abscess
    4. **Appendices**

## Appendix 1 - Patient information sheet

Please see version 2 of the following information sheets:

- - - - 1. Patient information sheet
        2. Day Surgery instruction sheet
        3. GP letter
        4. ED ward clerk letter

## Appendix 3 – Standard Powerchart forms/templates

- - - - 1. Abscess protocol form
        2. Abscess drainage operation report template

| Revision Date | Version | Author | Amendments |
| --- | --- | --- | --- |
| Nov 2017 | Version 2 | Tony Pang |  |
| 31/08/2018 | Version 3 | Tony Pang | Surgical Short Stay |
| 27/2/2019 | Version 3.1 | Tony Pang | Further clarification re short stay |
| 20/1/2022 | Version 4 | Tony Pang | Clarification |
